# Blood Glucose Level Prediction for Diabetics Based on Nutrition and Insulin Administration Logs Using Personalized Mathematical Models

**DOI:** 10.1155/2019/8605206

**Published:** 2019-01-10

**Authors:** Péter Gyuk, István Vassányi, István Kósa

**Affiliations:** ^1^Medical Informatics Research & Development Centre, University of Pannonia, Egyetem u. 10, 8200 Veszprém, Hungary; ^2^Department of Medical Rehabilitation and Physical Medicine, University of Szeged, Dugonics tér 13, 6720 Szeged, Hungary

## Abstract

According to recent surveys, the current ways of diabetics trying to estimate their insulin need based on experience and conjecture are sometimes inefficient in practice. This paper proposes a prediction algorithm and presents the validation of the model in outpatient care. The algorithm consists of two state-of-the-art models that calculate nutrition absorption and glycaemia including insulin evolution. The combined model is extended with personalized parameter training including genetic algorithm and Nelder–Mead method, and a more realistic, diurnal parameter profile as a representation of the natural biorhythm. This method implemented in a user-friendly application can help diabetics calculate their insulin need. The tests were performed on a data set including a clinical trial involving more than 20 diabetic patients. We experienced 55% improvement in the results due to model training compared to the tests based on literature parameters. In the best case, 92.5% of the predicted blood glucose level values were in the range of clinically acceptable errors, which means around 2.8 mmol/l root mean square error. The results of the validation based on outpatient data are promising compared to others found in the literature. Handling other important factors such as physical activity and stress remains a challenge for future research.

## 1. Introduction

Diabetes mellitus, a metabolic disease, is a crucial problem in modern health care, since it currently hits more than 8% of the adult population (age 20–79 years) [1]. Recent predictions report that this number can increase by 55% within 2 decades [1], which could also increase the mortality rate and the incidence of further complications caused by the disease. This underlines the importance of treatment of diabetics and finding new ways of diabetic lifestyle support.

As the current state of the art in medical science does not provide a full cure of the disease, the patients have to adopt a special lifestyle with a slightly different treatment for each type of diabetes; for some patients, it is enough to pay attention to the food intake while others need subcutaneous insulin injections to compensate the insufficient insulin production in their body. The two main types of the disease are type 1 diabetes (T1D) characterized by absolute deficiency of endogenous insulin production and type 2 diabetes (T2D) characterized by partial deficiency of endogenous insulin production and insulin resistance. This work concentrates on the daily life support of type 1 and type 2 diabetes outpatients treated with subcutaneous insulin injections. Subcutaneous insulin products have long-acting (or basal) types with a day-long effect, typically administered once a day in the morning or in the evening, and short-acting (or bolus) types administered separately for each main meal in order to control blood glucose level (BGL). The little mistakes in choosing the right dose of these injections can lead to a critically low BGL, that is, hypoglycemia, which may present an instant medical emergency, or a sustained, excessively high BGL, that is, hyperglycemia, which results in severe complications (e.g., cardiovascular diseases, kidney disease, and diabetic retinopathy) in the long run.

Estimating insulin needs before every meal and other activities is a daily task for each diabetic. These decisions are usually based on experience and conjecture, which is sometimes rather inefficient in practice, resulting in high glycated hemoglobin (HbA1c) values [2, 3]. These observations justify the idea of developing blood glucose prediction algorithms and lifestyle support applications that help diabetics in their everyday life [4, 5].

## 2. Materials and Methods

### 2.1. Objective

The aim of our work is to create an algorithm that is able to predict blood glucose evolution based on the personal nutrient intake and subcutaneous insulin injections data of diabetic patients using mathematical modeling. The final result would be a lifestyle mirror app including meal, medication, and other logging services with BGL prediction based on the log. Such a mobile application could help diabetics in their self-control, working for better HbA1c values. A lifestyle log application has been already developed by the Medical Informatics Research and Development Centre (MIRDC) of the University of Pannonia [6, 7], which can be the future host of the prediction module presented in this paper.

The main topic of this study is to combine, train, and extend BGL prediction models found in the literature. While most of these methods are developed for inpatient care, our aim is to make the models available for outpatient care by combining them with digestion modeling and new training techniques for a better parameter identification. In the current development phase of the prediction algorithms, the time span of the prediction is within a few hours (1–6 hours), and the input comes from a lifestyle log including meal intake and insulin dosing.

The BGL prediction model to be constructed should yield an accurate prediction close to the margin of error of the main stream BGL measurement devices and reliably predict hypo- or hyperglycaemia, that is, critically low or high BGL. Considering measurement device inaccuracy and previous results reported in the literature, a prediction method with an error range of 1–2 mmol/l root mean square error (RMSE) for short term, that is, 30–60 minutes, and 4–5 mmol/l for longer term, that is, 6-hour prediction, could already be potentially useful in practice.

### 2.2. State of the Art

There are several methods for BGL prediction found in the literature using different types of mathematical models and parameter identification methods [8], but a lot of these solutions ignore the effect of some important factors such as nutrient absorption variations or biorhythm. This section sums up the most recent and successful works that can be compared to our results.

The system used by Stahl and Johansson [9] consists of three parts that are separately modeled with compartment and linear black-box models. The system proposed by the authors does not model the digestion, but instead the carbohydrate intake dynamics was estimated for predefined meals. The BGL input data came from a type 1 diabetic patient using MiniMed Continuous Glucose Monitoring System (CGMS) during a 6-month period. In one of their other works [10], they used finite impulse response models on 18 patients to estimate postprandial plasma glucose level. For the evaluation, they used Clarke's Error Grid Analysis (EGA) [11].

Robertson et al. [12] demonstrated Elman's recurrent artificial neural network (ANN) based on meal and insulin intake. The data set originated from a free, artificial mathematical diabetes simulator called AIDA that modeled 28 days of measurements of a T1D patient. Regarding the meal intake, only carbohydrate quantities were considered, and the results are based on the quite limited food absorption modeling capabilities of AIDA [12].

Another, neural network-based solution is presented by Shanthi and Kumar [13]. The difference between their work and the previously mentioned ANN-based tests is that in this case, the validation data history included real patients in a hospital setting with different insulin therapies using Medtronic's CGMS.

The aim of the study reported by Plis et al. [14] is to avoid hypoglycemia during 30 minutes with BGL prediction. They used the support vector regression (SVR) and ARIMA models. The parameter identification was performed with an extended Kalman filter [15].

The method and the validation by Khaled et al. [8] carried out a 30-minute (short term) BGL prediction using genetic algorithms. The training data set was 1-hour long, and the training-validation ratio of the data is 2:1. The input of the validation came part from the AIDA simulator and part from volunteers. The meal intake for outpatients was modeled as a bolus injection of glucose.

There are other methods reported in the literature as well [16, 17], but they usually have more shortcomings as they use simulated input data or simple models that are less usable in outpatient care. The biggest shortage of the mentioned models is the lack of handling complex nutrient intake and glucose absorption. Thus, although there are promising solutions, the optimal setting is not yet found, and therefore our vision is to find or get closer to an even better model.

### 2.3. Proposed Method

Our method relies solely on nutrient consumption and insulin administration as input. Other factors like physical activity or stress are not included as input data in the model as no reliable algorithmic method was found in the literature that could handle their effects on BGL.

**Figure 1:**
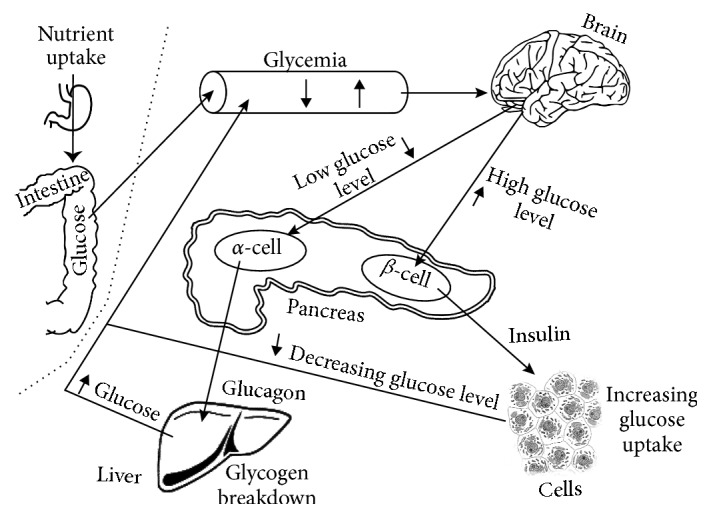
The process of metabolism.

Human metabolism can be divided into two major parts as shown in Figure 1. One of them is the main glucose control process including insulin appearance in the blood and the reaction mechanism to the changing BGL (*α*-, *β*-cell). This part is matched with the second subsystem including nutrient uptake and handling the resulting glucose absorption. These processes have slightly different characteristics for everybody, especially for diabetics. As the figure shows, the metabolism is a complicated process; therefore, we need complex models and a big parameter set to simulate real-life systems. Most of the previously published models are designed for inpatient care, usually with only glucose as meal input, so they need training and additional learning techniques to be applicable in outpatient care. This motivates us to prefer such models that have a big parameter set to effectively simulate each individual patient.

The proposed algorithm combines two published, state-of-the-art models to simulate the whole metabolism process [18, 19]. The fact that we use a complex model for the meal absorption and an advanced model of the glucose control system with many parameters makes our method applicable in outpatient care, which is a considerable advantage over other methods presented in the Introduction.

#### 2.3.1. Glucose Absorption Model

There are several methods that model the glucose absorption from meal intake [20]. Some of these models—such as the Diabetes Advisory System (DIAS) [21], which is a solid base of many others—include one-compartment algorithms modeling solely stomach absorption and taking some important components (e.g., lipids and proteins) out of consideration. Other, more complex and improved, two-compartment algorithms, like the one we chose [18], give a better representation of the real body processes with added features, more parameters, and introducing the intestine compartment.

**Figure 2:**
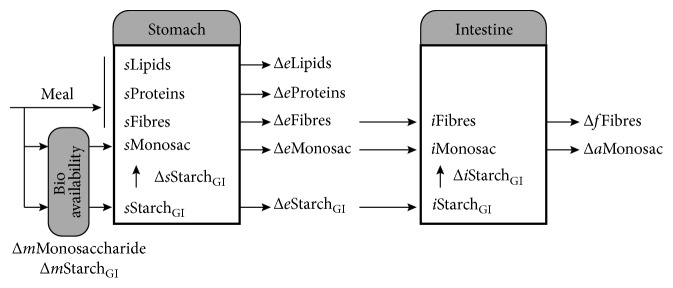
The structure of the glucose absorption model.

The overview of the chosen model [18] is shown in Figure 2. The choice was motivated by this method having an extended parameter set including lipids, proteins, fibers, monosaccharides and starch with different glycemic indices (GI) [22]. This feature ensures the correct representation of mixed meals as it can distinguish meals with different absorption characteristics taken at the same time. The big parameter set helps manage the complex nutrients close to reality. Another important feature of this model is its support for overlapping nutrient evolutions that happen when the absorption caused by the first meal is still in progress at the time of the following meal intake. The model uses simple mass balance equations to simulate the whole digestion system divided into two compartments. A detailed description of the equations can be found in the original article [18].

#### 2.3.2. Glucose Control and Insulin Absorption System

The other important part of the combined model is the glucose control system that calculates the insulin evolution. A great overview about these methods is presented in [23]. Many of these algorithms are based on the original minimal model [24], which is a stable base of BGL estimation, but lacks in parameter set and model complexity, resulting in weaker prediction force. Other, more sophisticated methods include integro-differential [25], partial differential [26], and delay differential equations (DDE) [19]. A common feature of these models is that they have been validated and developed for inpatient care, while this study validates them in outpatient care with many other factors that the solutions do not take into consideration. Some of the very sophisticated, complex models give a good representation of the insulin evolution process, but due to their overly extensive parameter set, they are very hard to identify and, therefore, to apply, in practice.

The chosen model is created by Palumbo et al. [19] and based on DDE. The choice was motivated on the relatively small set of parameters and the still powerful descriptive capabilities (e.g., support for two subcutaneous depots) compared to the very simple models like the Minimal. The main equations of the model (1–4) are as follows:

(1) $$\begin{array}{c} \frac{dG}{dt}=-K_{xgi}\;*G\left(t\right)\;I\left(t\right)+\frac{T_{GH}}{V_{G}}, \end{array}$$

(2) $$\begin{array}{c} \frac{dI}{dt}=-K_{xi}\;*\;I\left(t\right)+\frac{T_{iG\max}}{V_{I}}\;*\;f\left(G\left(t-\tau_{G}\right)\right)+\;\frac{1}{V_{I}\;*\;t_{\max,I}}\;*\;S_{2}\left(t\right), \end{array}$$

(3) $$\begin{array}{c} \frac{dS_{2}}{dt}=\frac{1}{t_{\max,I}}\;*\;S_{1}\left(t\right)-\frac{1}{t_{\max,I}}\;*\;S_{2}\left(t\right), \end{array}$$

(4) $$\begin{array}{c} \frac{dS_{1}}{dt}=-\frac{1}{t_{\max,I}}\;*\;S_{1}\left(t\right)-u\left(t\right). \end{array}$$

The first equation calculates the BGL (*G* (mmol/l)), while the second one models the insulin evolution in the body (*I* (pmol/l)). Equations (3) and (4) describe the two subcutaneous depots that simulate the real insulin absorption process after subcutaneous insulin injection. Function *u*(*t*) describes the insulin input, while the *f*(*G*(*t* − *τ*_*G*_)) function used in Equation (2) represents the endogenous insulin production (Equation (5)):

(5) $$\begin{array}{c} f\left(G\right)=\frac{{\left(G/G\right)}^{\gamma }}{1+{\left(G/G\right)}^{\gamma }}. \end{array}$$

To combine this model with the glucose absorption algorithm, a little change had to be made in Equation (1). As a result, Equation (6) contains the monosaccharide absorption through the intestine wall (∆*a*Monosac(*t*)) calculated by the digestion model.

(6) $$\begin{array}{c} \frac{dG}{dt}=-K_{xgi}*G\left(t\right)*I\left(t\right)+\frac{T_{GH}}{V_{G}}+\Delta a\text{Monosac}\left(t\right). \end{array}$$

The description and optimized values of the parameters are shown in Table 1. For a more detailed description of the model, see [27].

#### 2.3.3. Parameter Identification

Table 1 shows the typical values of the model parameters. However, the personal variations can significantly differ from the above values. Though most of the personalized values could be determined by a CGMS-based clinical glucose tolerance test [27], such a test is not typically performed in outpatient care due to its high costs, and only a single measurement is taken at 90 or 120 minutes. Since such significant uncertainties would make the model outcome practically useless, we need personalization, that is, parameter training. Obrączka and Mitkowski present a great overview of the parameter identification methods in [28]. We compared several of these methods to find the right combination that best matches our problem domain.

Since diabetes is not correlated with digestion disorders, we can assume that the absorption model parameters are the same for everybody and we focus on the 9 parameters of the glucose control system shown in Table 1. In order to find the most important parameters, we made a one-factor-at-a-time (OFAT) sensitivity analysis using 15 data sets including more than 200 meals and around 80 days of diverse outpatient data.

The first parameter identification method that we used to train the model is the simple brute force (BF) method. A setup of 7 steps for each parameter has been used with 20% step size, which has 343 iteration for the 3 main parameters (*K*_*xgi*_, *K*_*xi*_, and *V*_*i*_).

The other method that we used is the genetic algorithm (GA) [29, 30], which is a stochastic algorithm; hence, subsequent runs have different outcomes with the same options and input data, which is why other numerical training methods are usually employed to refine the solution. The parameters of the genetic algorithm include mutation and crossover probability, population size, and generation (iteration) number.

Finally, we implemented a simplex method to fine-tune the trained parameter set delivered by the GA. The Nelder–Mead downhill simplex method has been used, which is an ideal method to minimize functions with more variables [31]. We did not use this as a separate training method because it proved to be much less effective than the GA or the BF method.

#### 2.3.4. Support for Diurnal Parameter Variations

**Figure 3:**
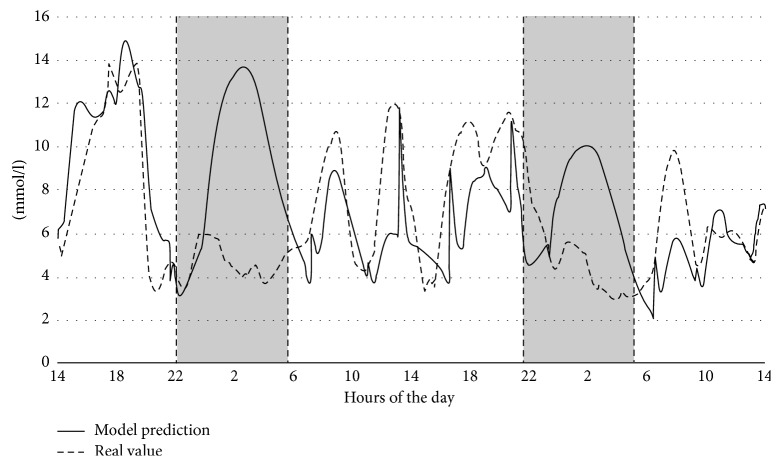
Nightly prediction error patterns for a patient. Measured BGL are shown as a dashed line, and night periods are shaded in grey.

The characteristics of the human glucose system are known to change according to a diurnal pattern [32]. Both insulin sensitivity and secretion is supposed to change in the evening [33], but diabetics may also experience differences between early morning and afternoon hours. As Figure 3 shows an example model outcome versus measured BGL for two days of a patient, the most severe errors, shaded in grey in the figure, occur typically during nighttime due to a change in insulin sensitivity not handled by the model. This effect may combine with the basal insulin administered at bedtime. Though none of the models presented in the literature overview support this variation, we implemented the above parameter identification scheme such that we find the personalized parameter values for each distinct period of the day. The diurnal parameter profiles are then computed from the values found for the periods interpolated by spline interpolation to form a smooth curve.

Insulin sensitivity is of course just one of the parameters of the model, and, theoretically, other parameters could also have a systematic diurnal variation. However, it was the relevant clinical literature [32] and our own practical experience regarding the varying effects of a certain dose of insulin during the day, which suggested us focusing solely on this single parameter.

#### 2.3.5. An Overview of the Validation Clinical Study

Our model was validated with a clinical study, performed at the Cardiac Rehabilitation Institute of the Military Hospital, Balatonfüred, Hungary. The study included insulin-dependent type 2 diabetes patients taking part in 3-week courses of rehabilitation with daily activities similar to everyday life, yet under continuous medical supervision, which presented an ideal environment for testing the model. The patients logged every meal and applied dose of insulin and every value of self-measured blood glucose along with any physical training activity performed in the Lavinia Lifestyle Mirror mobile application running on mobile phone (Nexus 5, LG Electronics, Seoul, South Korea), or tablet (Nexus 7, AsusTek Computer Incorporation, Taipei, Taiwan) during their 21-day hospitalization period. Blood glucose self-control was performed with fingertip devices as clinically indicated and the patient's metabolic status was performed by a CGMS (Guardian Pro, Medtronic, Northridge, CA). The tests were run between 14 January 2015 and 5 April 2015.

#### 2.3.6. Ethical Considerations

The study protocol was approved on 18 October 2013 by the institutional ethical committee of the Military Hospital, Budapest, Hungary, chaired by Dr. László Kovács, under the submission number II/20-265-2013. The protocol was designed and implemented in compliance with the World Medical Association Declaration of Helsinki on Ethical Principles for Medical Research Involving Human Subjects.

### 2.4. Data Used for Validation

The input data consisted of the lifestyle logs (meals and insulins) and CGMS BGL readings sampled at 5 minutes of 26 type 1 and type 2 diabetic patients (15 men and 11 women, average age of 62.85). The full data set consisted of 30 different data files including 142 days of nutrition, medication logs, and BGL measurements with each log consisting of at least 3 days of logging. There were 3 patients who had more than 6 days of data file; these were divided into 7 new ones in total. This means almost 600 meals and subcutaneous insulin inputs with 8 types of popular bolus and 6 types of basal insulin types, and a total of around 40000 CGMS or manual BGL measurement values.

#### 2.4.1. Data Processing Methods

For the logging of the meals and insulin administration of patients, paper-based forms and the Lavinia Lifestyle Mirror mobile application were used [7]. All data were stored in a Postgres 9.4 database and analyzed with a custom-built client tool written in the C++ language. Electronic logs were manually compared to paper logs and were classified according to the type and quantity of errors. From the original 34 data files, 4 had to be discarded due to fully inconsistent logging, using insulin pumps or oral medication instead of insulin injections.

## 3. Results and Discussion

**Figure 4:**
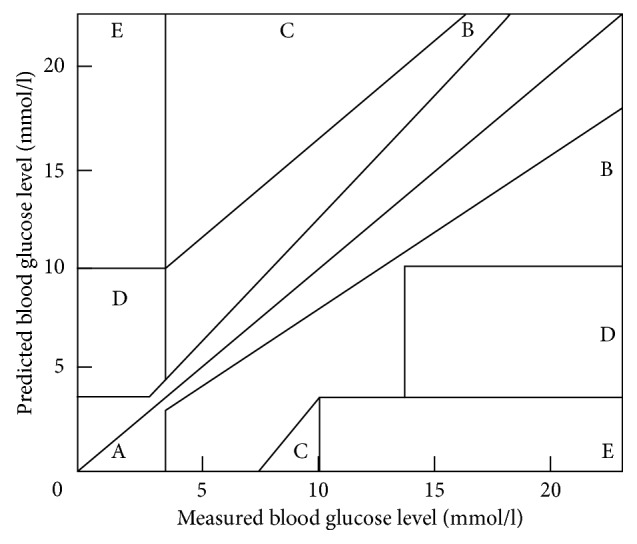
The Error Grid Analysis assessment ranges for BGL prediction.

The validation process consisted of several phases that probed the parameter training methods described above. In this section, we specify the results of each phase. In order to assess the quality of the prediction, we used several figures of merit. Besides the absolute total, average, maximal error, variance, and RMSE, we also used Clarke's Error Grid Analysis (EGA) as a popular BGL prediction algorithm validation method [11]. The EGA classification of a BGL prediction uses A, B, C, D, and E classes, based on the severity of the expected clinical effect of a clinical decision, for example, insulin dosing, based on a predicted BGL (Figure 4). In this respect, the most severe error with the worst classification of D or E is an overly high BGL prediction in the <4 mmol/l actual range, for example, a predicted 7 mmol/l versus an actual (real) BGL of 3 mmol/l because it can lead to hypoglycaemia. The same 4 mmol/l error is classified in the “clinically acceptable” A or B class if a BGL of 12 mmol/l is underestimated as 8 mmol/l. In short, while RMSE is an indicator of the achieved precision of the model, EGA shows the clinical applicability of the result. The improvement in the results was supported by the two-sample *t*-test with a significance level of 5%.

### 3.1. Identifying the Most Sensitive Model Parameters

**Figure 5:**
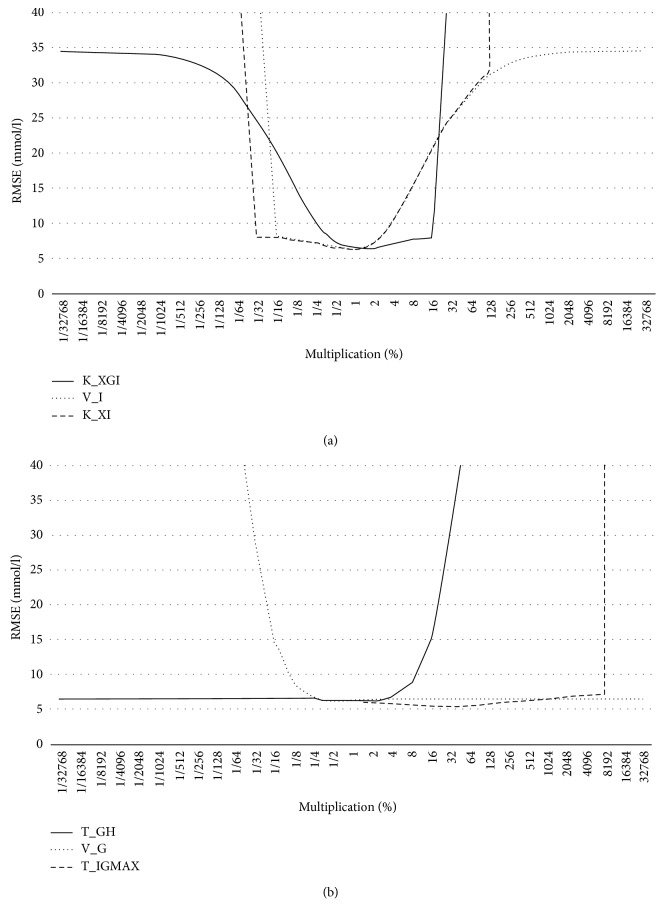
Model sensitivity analysis: 6 parameters that have the most significant effect on the model outcome (RMSE) depending on the value change of the parameter (multiplication).

The OFAT test showed that the 3 parameters *K*_*xgi*_, *K*_*xi*_, and *V*_*i*_ have the biggest impact on the BGL predicted by the model, and there are 3 more parameters that have remarkable connection with the outcome of the model; these are *T*_*GH*_, *V*_*G*_, and *T*_*iG*max_ (Figure 5). The remaining 3 variables (*G*^*∗*^, *τ*_*G*_, *γ*) have only a minor effect on the results.

### 3.2. Diurnal Parameter Profiles

**Figure 6:**
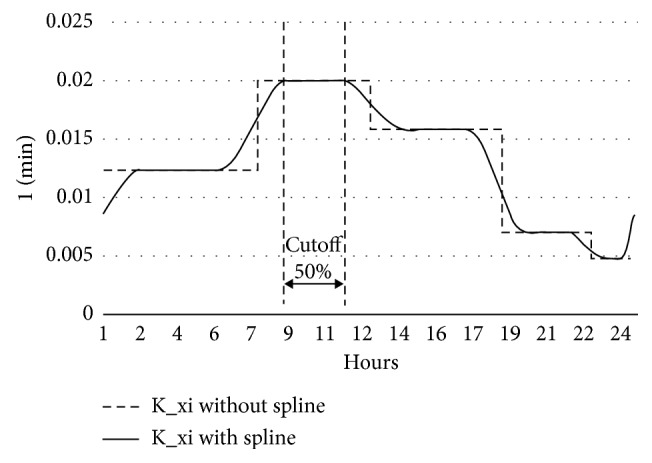
An example parameter profile with four trained parameter values. The solid line shows the implemented profile.

In order to implement the diurnal parameter profiles, we first had to define the time periods of the profile. Practicing diabetologist experts recommended us at least three distinct sections of the day. After trying several algorithmic setups and consulting with a diabetologist, we found that the best prediction is ensured when we use four equal-length distinct periods of the day, that is, those of 0:00–6:00, 6:00–12:00, 12:00–18:00, and 18:00–0:00.  Figure 6 shows a concrete example of a patient's *K*_*xi*_ parameter profile with the four trained values smoothed with a spline.

### 3.3. Model Restart for Realistic Evaluation

**Figure 7:**
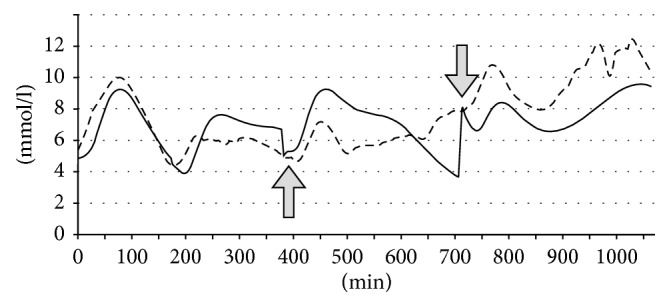
The effect of restarting the model at fingerstick BGL measurements (continuous line: model prediction; dashed line: CGMS measured value). Arrows point at model restart events.

A diabetic patient on an insulin regime routinely measures her BGL with a fingerstick device before each main meal before setting the bolus insulin dose for the meal. We had access to these manual measurement data in the patient's log. In a realistic scenario, a lifestyle support application can also deploy this information to correct the cumulative errors of the BGL prediction by restarting the underlying model at each manual measurement. As a first step of the validation, we implemented a model restarting scheme in the prediction algorithm such that we set the current BGL to the measured value, but we do not change other state variables like the *S*1, *S*2, and *I*. In this way, we still correctly model digestion and insulin evolution processes that overlap between two consecutive meals. As Figure 7 shows with data taken from a log, a restart can effectively decrease the error of the model.

Table 2 shows numeric test results with and without restart and without any parameter training or diurnal profiling. The table also shows the 1-, 2-, 4-, and 6-hour “meal-wise” test results in the first columns. During these meal-wise tests, the model was started at meal times and run for 1, 2, 4, and 6 hours without considering any previous insulin inputs or meal intakes. Therefore, the unhandled effect of overlapping digestion and insulin evolution is expected to cause significant errors. The “full data” set in Table 2 refers to all logs of all 26 patients. For an analysis of the results in the tables, see Discussion.

### 3.4. Parameter Training Methods

The BF method was used with 7 steps and 20% step size. Though this is not the closest setting to reach optimal results, further refinement (more steps with smaller step size) do not result in significantly better outcome compared to the negative effect of considerably longer running time.

**Figure 8:**
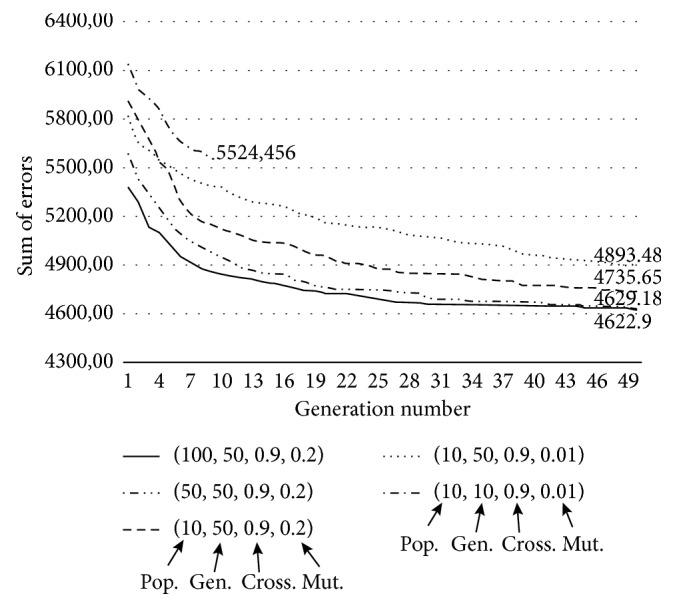
Algorithm parameter test. The 5 different parameterizations are shown as how they affect the sum of errors of the model prediction using the trained model parameters.

Regarding the GA, we performed an extended test on the same 15 data sets that were used for the model sensitivity analysis (Figure 8) and reached the limit of this algorithm with population size and generation number of 50, crossover probability of 90%, and mutation probability of 20%, making this method a slower but more efficient alternative of the brute force search algorithm. An interesting observation is that increasing the population size to 100 (continuous line) has not produced better results after 50 generations, which marked a reasonable bound for these parameters. In the followings, we used two different parameterizations of the algorithm, that is, the original version with a population and generation number of 10, a crossover probability of 90%, and a mutation probability of 1% and the algorithmic setup described before. From Table 3 on, we will refer to the original version as the “Fast GA” and the version with the increased population sizes as the “Slow GA.”

The main parameter identification methods were validated in Phase 2, in which several running scenarios were tested with different fitness functions. The brute force and genetic algorithm methods were used to train the 3 main parameters (*K*_*xgi*_, *K*_*xi*_, and *V*_*i*_). Table 3 shows the three main figures of merit for the full data set.

### 3.5. Diurnal Profiling and Extended GA Parameter Set

After these steps, we introduced the diurnal profiles and measured the effects of the GA refinement, that is, slower, but more efficient parameterization version of the GA with population size and a generation number of 50, crossover probability of 90%, and mutation probability of 20%. The results are shown in Table 4.

### 3.6. Extended Model Parameter Set and Downhill Simplex Method

In Phase 4, we examined the result of increasing the set of trained model parameters from 3 up to 6 and 9 parameters. As we got the best results with 6 parameters (*K*_*xgi*_, *K*_*xi*_, *V*_*i*_, *T*_*GH*_, *V*_*G*_, and *T*_*iG*max_), this scenario was used for the test of downhill simplex method together with GA. The iteration number of the Nelder–Mead method was set to 100, which resulted in a running time similar to the GA itself. Results are summarized in Table 5.

### 3.7. Using the Trained Model for Prediction

The practical application of the trained model is to provide reliable short-time predictions in a lifestyle support application. In order to assess the predictive power of the model, we finally used a 3-day-long sample of each patient's log to implement a cross-validation scheme. A 1-day wide sliding window was used to train the personalized parameters with our best algorithmic setup, that is, GA with 6 parameters, downhill simplex plus diurnal profiles, and the rest of the data were used to validate the model. By sliding the window to 10 distinct positions with a 3-hour step size, we performed 10 such validations and took the average of the computed 1-hour meal-wise RMSEs. Then, we repeated the test with a 2-day wide sliding window for training data. The 1-day and 2-day results are shown in the first and second row of Table 6, respectively.

## 4. Discussion

First of all, it is important to note that according to clinical tests, the CGMS has an error range with a median average difference of 1.4 mmol/l and a relative average difference of 17% in outpatient care [34]. These values, measured on young type 1 diabetics, are close to those published in the manual of the device. Moreover, according to the graphs of the manual, the CGMS has difficulties measuring high and low peaks, often resulting in significant over- and underestimations that are getting worse towards the end of the six-day lifetime of a CGMS sensor. These factors should be considered when assessing the outcome of the model as the correction of this phenomenon is yet a plan for the future.

The test on the full data with model restart delivers around *10–15% improvement* depending on the figure of merit (Table 2). The average error has decreased to 4.37 mmol/l from 5.09 mmol/l, which is caused by the elimination of cumulative errors. Comparing the meal-wise tests with the long-time data runs, the advantage is clear for the good of the full test with restart (*20+%*), excluding the 1-hour prediction, where the prediction resulted in 3 mmol/l RMSE. This shows that for longer term, the handling of the overlapping glucose and insulin absorption processes, and also the effect of the long-acting insulin evolution is very important regarding the outcome of the model.

The parameter identification methods (Table 3) result in almost *20% improvement*. The fact that the two training methods delivered nearly the same results shows the power of the genetic algorithm as it had a faster running time than the brute force method. Based on the numbers, besides the 3.64 mmol/l average error, we can state that with the model training, *in 83% of the time*, the GA gives clinically acceptable results. Regarding the diurnal parameter profile (Table 4), we observed a significant *improvement of 5–10%*, resulting in a *3.3 mmol/l average error*, which shows the importance of representing the changing biorhythm in the model.

The biggest improvement was achieved by the extension of the training parameter set (Table 5) and by using more effective parameterization of the GA. Compared to the faster version of GA, the slower version of the algorithm delivers ca. 10–15% improvement as the average error decreases *under 3 mmol/l*. The extension from 3 to 6 parameters brought a *huge improvement of 25–30%*; however, the set with 9 parameters resulted in worse numbers. This shows that the best solution is to use 6 parameters for the identification (*K*_*xgi*_, *K*_*xi*_, *V*_*i*_, *T*_*GH*_, *V*_*G*_, and *T*_*iG*max_), and the other parameters are better be used with the optimized literature values. Introducing the Nelder–Mead algorithm, the average error decreased with an additional 5%, reaching our best results, where the prediction is *clinically acceptable in 92.5% of the time* with *1.98 mmol/l average error* and *2.76 mmol/l RMSE* for a continuous, multiple-day time interval. This improvement by the Nelder–Mead method is not too effective considering the long running time, but other numerical methods that use derivatives could mean an upgrade in the future.

Finally, the prediction model validation showed that the most effective GA trained on a 2-day sample can predict BGL for 1 hour with an error of *1.62 mmol/l RMSE*.

### 4.1. Evaluation

Comparing our results with others found in the literature, the model seems promising as the numbers are close or sometimes even better than the outcome of other models developed for, and tested in, inpatient care. Stahl et al. [9] used CGM and stayed in the A error zone of EGA in 75% of the time for 1-hour prediction, while we reached around 60% for the same time period.

At the same time, Robertson et al. [12] performed a 1-hour prediction resulting in errors under 0.27 mmol/l with 0.15 mmol/l RMSE. These figures seem really convincing, but we must keep in mind that the validation was based on a data set provided by a simulator that does not model personal variations of the BGL control system and other important factors of real life such as stress or physical activity.

The validation by Shanthi and Kumar [13] that includes real patient input data in a hospital setting shows a slightly worse outcome as the best result of their method is 0.78 mmol/l RMSE during a 60-minute-long prediction. The disadvantage is that the ANN was trained only using the BGL curve without meal absorption. Plis et al. [14] reached 1.99 mmol/l RMSE for the same period with similar input data and validation process. As we reached 1.62 mmol/l RMSE for the 1-hour prediction with 2 days training data set using real data, it shows there is still room for improvement, but we should not forget that although these presented models took meal absorption into account, they only rely on carbohydrate input.

The method and the validation by Khaled et al. [8] is the most relevant article to compare with the results of this paper regarding the prediction method as they carried out a 30-minute (short term) BGL prediction using genetic algorithms. The input of their validation, however, came partly from the AIDA simulator, which is a little setback in regards of a good comparison with our results as they worked with virtual patients in more than half of the cases. Another shortcoming is that the meal intake for outpatients was modeled as a bolus injection of glucose. The RMSE value was 0.5 mmol/l during the 30-minute validation with the 1-hour training set. It should be considered that this is a really short-term prediction without a wide nutrient operational range.

A known limitation of our approach is the selected BGL control model itself, as it may oversimplify the very complex mechanisms of the BGL control system. More elaborate models that are closer to the clinical reality are also available, but at the expense of a much higher number of parameters, making the model very hard to train for a certain patient. The parameters of the model applied in our work all have a concrete physiological meaning and (theoretically) they could all be measured for a certain patient in a clinical setting, via complex medical protocols. In our clinical trial, we did not have access to such facilities, so while we excluded clinically *impossible* parameter values, the only proof on that we found the “right” values is that the identified parameter set was found to minimize the error of the prediction. This note also refers to the credibility of the postulated diurnal variations, exemplified in Figure 6—while variations of such magnitude are clinically possible, the actual values could be verified only in a clinical setting.

It should also be noted that even the best RMSE 1.62 mmol/l achieved in our work means no guarantee on that the predicted value always leads to clinically acceptable (EGA A or B) decisions, not even for predicted values above 5 mmol/l since the distribution of the error being close to the normal distribution with zero mean, even an error in the range of 3 mmol/l may occur in a low number (>2%) of the cases. The interpretation of EGA is quite different because unlike the RMSE, it is not symmetrical, it penalizes certain types of errors more, and, therefore, we cannot compute an equivalent RMSE value for an EGA percentage measured on a certain data set.

## 5. Conclusions

The paper presented a BGL predicting method that is based on the combination of accepted absorption and BGL control models, thus allowing its application for diabetic outpatients keeping a mobile lifestyle log. We trained the parameter set of the BGL control model in order to provide a reliable, personalized prediction. The results show that 6 parameters trained with genetic algorithms in diurnal profiles using 2 days of CGMS training data deliver an RMSE as low as 1.62 mml/l in 1-hour predictions and clinically acceptable results in more than 90% of the cases. This proves the practical applicability of the method if the results of our small-scale validation trial could be verified in a larger study.

There are several open questions for future research. The personalized parameters may change over time, or seasonally, but we cannot expect to have access to CGMS training data all the time, so the model should be retrained adaptively using the logged fingertip records. The known errors and dynamics of the CGMS device could be integrated in the training process. Also, the model itself could be refined to properly handle the action of basal insulin, which is now implemented as a bolus insulin with a long action. We feel that these extensions together with the proper handling of the physical activity and stress factor could be tracked easier with a model-less control method (such as deep neural networks). Treating these as additional input parameters could be a quick and efficient solution if the training data set grows sufficiently large.

## Figures and Tables

**Table 1:** Glucose control model parameters (kgBW = body weight in kilograms).

Name	Description	Unit	Value^*∗*^
*K*_*xgi*_	Rate of glucose uptake by insulin-dependent tissues	1/(min *∗* pM)	3.11 *∗* 10^−5^
*T*_*GH*_	Net balance between hepatic glucose output and insulin-independent zero-order glucose uptake by brain	mmol/(min *∗* kgBW)	0.003
*V*_*G*_	Apparent distribution volume for glucose	*L*/kgBW	0.187
*K*_*xi*_	Apparent first-order disappearance rate constant for insulin	1/min	1.211 *∗* 10^−2^
*T*_*iG*max_	Maximal rate of second-phase insulin release	pmol/(min *∗* kgBW)	0.1
*V*_*i*_	Apparent distribution volume for insulin	*L*/kgBW	0.236
*τ*_*G*_	Apparent delay with which the pancreas varies secondary insulin release in response to varying plasma glucose concentrations	min	24
*t*_max,*I*_	Time-to-maximum insulin absorption	min	Insulin product dependent
*G* ^*∗*^	The glycaemia at which the insulin release is half of its maximal rate	mmol/l	9
*γ*	The progressivity with which the pancreas reacts to circulating glucose concentrations	—	3.205

^*∗*^Optimized values from [27].

**Table 2:** Validation results with literature parameters (values in mmol/l).

Tests with parameters from the literature	Meal-wise test				Full data test	
	1 h	2 h	4 h	6 h	Without restart	With restart
Average error	2.22	5.47	7.80	8.00	5.09	4.37
RMSE	3.00	6.79	9.10	9.44	6.54	5.72
EGA − A + B (acceptable)	89%	67%	56%	57%	73%	78%

**Table 3:** Validation results for the full time period with various algorithmic setups as indicated (values in mmol/l).

Model training (full data test with restart)	Without training	Brute force	Fast GA	Fast GA + diurnal parameter profile
Average error	4.37	3.57	3.64	3.30
RMSE	5.72	4.74	4.82	4.43
EGA − A + B (acceptable)	78%	84%	83%	85%

The error is the difference of the CGMS reading and the model outcome.

**Table 4:** Results for the full time period with diurnal profile and various GA versions (values in mmol/l).

Diurnal profile and GA test with 3 model parameters (full data with restart)	Average error	RMSE	EGA − A + B (acceptable)
Fast GA without diurnal profile	3.64	4.82	83.2%
Fast GA with diurnal profile	3.25	4.54	86.5%
Slow GA with diurnal profile	2.94	4.09	88.6%

**Table 5:** Results for the full time period with extended model parameter set and Nelder–Mead downhill simplex method (values in mmol/l).

Extended parameter set and Nelder–Mead algorithm test (full data with restart)	Average error	RMSE	EGA − A + B (acceptable)
Slow GA with 3 parameters	2.94	4.09	88.6%
Slow GA with 6 parameters	2.11	2.96	92.2%
Slow GA with 9 parameters	2.16	3.05	92.1%
Slow GA with 6 parameters + Nelder–Mead method	1.98	2.76	92.5%

**Table 6:** Prediction model errors, RMSE values in mmol/l for 1-hour prediction.

Prediction model (RMSE, meal-wise)	Without training	Brute force	Slow GA
(1) day training cross validation	2.57	2.57	2.26
(2) days training cross validation	2.29	2.19	1.62

## Data Availability

The detailed dietary and CGMS log data used to support the findings of this study may be released upon application to the Institutional Ethical Committee of the Military Hospital, which can be contacted at Magyar Honvédség Egészségügyi Központ Intézményi és Regionális Kutatásetikai Bizottság, Róbert Károly körút 44, 1134 Budapest, Hungary.
